# Analysis of structure and dynamic characteristics for an electric tractor platform

**DOI:** 10.1038/s41598-026-50055-7

**Published:** 2026-07-23

**Authors:** Seung-Min Baek, Min-Jong Park, Hyeon-Ho Jeon, Wan-Soo Kim, Yeon-Soo Kim, Yong-Joo Kim

**Affiliations:** 1 Eco-friendly Hydrogen Electric Tractor & Agricultural Machinery Institute, Chungnam National University, Daejeon, 34134 Republic of Korea; 2 Department of Smart Agriculture Systems, Chungnam National University, Daejeon, 34134 Republic of Korea; 3 Department of Smart Bio-Industrial Mechanical Engineering, Kyungpook National University, Daegu, 41566 Republic of Korea; 4 Upland Field Machinery Research Center, Kyungpook National University, Daegu, 41566 Republic of Korea; 5 Department of Bio-Industrial Machinery Engineering, Pusan National University, Miryang, 50463 Republic of Korea; 6 Department of Smart Agriculture Systems Machinery Engineering, Chungnam National University, Daejeon, 34134 Republic of Korea

**Keywords:** Engineering, Materials science

## Abstract

The electrification of agricultural tractors introduces significant changes in vehicle architecture, mass distribution, and structural load paths. In particular, the transition from conventional diesel tractor platforms employing engine–axle integrated load-bearing structures to electrified configurations based on modular frame architectures requires a systematic evaluation of platform-level structural performance. This study presents a structural assessment of a modular electrified tractor platform through integrated analyses of structural strength, static stiffness, and free vibration characteristics. A fully integrated finite element model representing the electrified tractor platform was evaluated under representative worst-case loading conditions, including impact, braking, and implement-induced working loads. In addition, the evolution of dynamic characteristics was investigated through comparative modal analysis between progressively integrated electrified tractor platform models and a reference diesel tractor platform of the same power rating employing an engine–axle integrated structural architecture. The results reveal localized stress concentrations under severe impact- and braking-dominated load cases; however, the overall frame structure satisfies structural safety requirements for typical agricultural operating conditions. The stiffness evaluation confirms that the modular frame architecture provides sufficient resistance to global bending and torsional deformation despite the absence of a conventional engine block acting as a primary load-bearing component. Modal analysis indicates reduced dominant natural frequencies due to increased structural mass associated with electrification, while the primary vibration modes remain sufficiently separated from typical excitation sources encountered during agricultural operation. Overall, the findings demonstrate the structural feasibility of modular electrified tractor platforms and provide a structural design basis for future development of electrified agricultural machinery.

## Introduction

With growing concerns over environmental sustainability and greenhouse gas emissions, electrification has gained increasing attention as an alternative to conventional fossil fuel–based propulsion systems across various industrial sectors^1–4^. In the field of agricultural machinery, electric tractors have emerged as a promising solution to reduce emissions while maintaining functional performance for field operations^5–7^. Major global manufacturers have recently introduced battery-powered electric tractors, particularly in low- to mid-power categories, demonstrating the technical feasibility of electrified agricultural platforms^8, 9^. Unlike conventional internal combustion engine (ICE) tractors, electric tractors rely on battery-based propulsion systems in which engines are replaced by electric motors, power electronics, and energy storage systems^10–11^. Conventional ICE tractors have traditionally adopted engine–axle integrated structural configurations, in which the diesel engine and drivetrain components are mounted on the axle assembly and function as primary load-bearing elements of the tractor platform^13, 14^. This fundamental change in powertrain architecture significantly alters vehicle layout, mass distribution, and load paths^15, 16^. As electrification replaces the engine-based load-bearing concept with distributed electric components and high-capacity battery systems, the overall system mass increases and the structural load transfer mechanisms are substantially modified. As a result, electric tractors are increasingly developed using frame-type or modular structural architectures rather than traditional engine-based load-bearing configurations. These structural changes necessitate careful evaluation of structural safety, stiffness, and dynamic characteristics to ensure reliable operation under agricultural working conditions^17, 18^. The integration of large battery packs and distributed electric components requires new frame architectures capable of supporting increased structural loads while maintaining sufficient stiffness and durability^19^.

To evaluate such structural changes, numerical modeling techniques have been widely adopted to analyze the structural behavior of transportation systems under complex loading conditions^20–22^. Finite element (FE) analysis has become a standard approach for evaluating structural strength, stiffness, and durability of vehicle platforms during the design stage. In the railway sector, high-fidelity FE models have been developed to investigate the static and dynamic behavior of lightweight vehicle structures, including aluminum–honeycomb hybrid car bodies and composite sandwich panels, enabling detailed evaluation of structural safety and modal characteristics^23–25^. Similarly, lightweight structural concepts based on composite materials have been explored to reduce structural mass while maintaining sufficient stiffness and durability^26^. Beyond railway applications, numerical simulations have also been extensively applied to off-road and agricultural vehicles. Structural optimization of agricultural machinery components and off-road vehicle chassis has been conducted using finite element methods to evaluate stress distributions and structural reliability under representative working loads^27, 28^. In addition, advanced numerical techniques such as multi-body dynamic simulations and coupled DEM–FEM approaches have been proposed to analyze vehicle dynamics and tire–terrain interactions for off-road vehicles operating on deformable terrain^29, 30^. These studies demonstrate the growing importance of high-fidelity numerical modeling for understanding the structural and dynamic behavior of complex vehicle platforms. In modal analysis studies, the modal assurance criterion (MAC) has been widely adopted as a quantitative indicator for evaluating the consistency between numerical and experimental mode shapes, enabling reliable validation of FE models across various engineering applications^31^. These studies demonstrate the growing importance of high-fidelity numerical modeling for understanding the structural and dynamic behavior of complex vehicle platforms.

Despite the extensive application of numerical modeling techniques in various transportation systems, their application to the structural analysis of electrified agricultural tractors remains relatively limited. Previous research on electric tractors has largely concentrated on powertrain performance, energy efficiency, and operational characteristics under agricultural working conditions^32–34^. Numerous studies have examined motor and battery performance, energy consumption, and drivetrain control strategies using simulation and experimental approaches, demonstrating the potential of electrified powertrains to improve efficiency and controllability in agricultural applications^35–37^. Other studies have investigated the operational behavior of electric tractors under varying load conditions, highlighting the influence of driving speed, torque demand, and control parameters on overall system performance^38, 39^.While these studies provide valuable insights into the energy utilization and functional performance of electric tractors, their scope has remained primarily focused on propulsion systems and operational efficiency. In contrast, relatively limited attention has been given to the structural behavior of electric tractor platforms, particularly in terms of structural strength, stiffness adequacy, and vibration characteristics. Given that electrification introduces increased system mass and a shift away from engine–axle integrated load-bearing structures, a systematic structural evaluation of alternative frame architectures becomes essential to ensure structural safety, durability, and practical applicability. From a structural perspective, tractors are subjected to complex loading environments that include impact loads from uneven terrain, braking-induced loads, and working loads transmitted through rear-mounted implements. These loading conditions can lead to localized stress concentrations, global deformation, and vibration-related durability issues. Although prior studies have examined structural responses of vehicle frames under static and dynamic conditions, most have focused on on-road vehicles or simplified loading scenarios. For electric tractors, structural evaluations that reflect realistic agricultural operating conditions and electrification-induced structural changes are still required^40^.

Therefore, this study aims to evaluate the structural performance of a modular frame-based electrified tractor platform through an integrated assessment of structural strength, static stiffness, and free vibration characteristics. A fully integrated electric tractor platform model is analyzed under representative worst-case loading conditions to assess structural safety and stiffness adequacy. In addition, the evolution of dynamic characteristics is examined through comparative modal analysis between a reference diesel tractor platform employing an engine–axle integrated structural architecture and the proposed modular platform of the same power rating. By synthesizing the results of strength, stiffness, and modal analyses, this study seeks to verify the structural feasibility of modular electrified tractor platforms and to clarify the influence of electrification-induced architectural changes on static and dynamic structural behavior. The findings provide a structural basis for electric tractor platform design and serve as a foundation for future experimental validation using full-scale prototypes.

## Methods

### Electric tractor platform

**Fig. 1:**
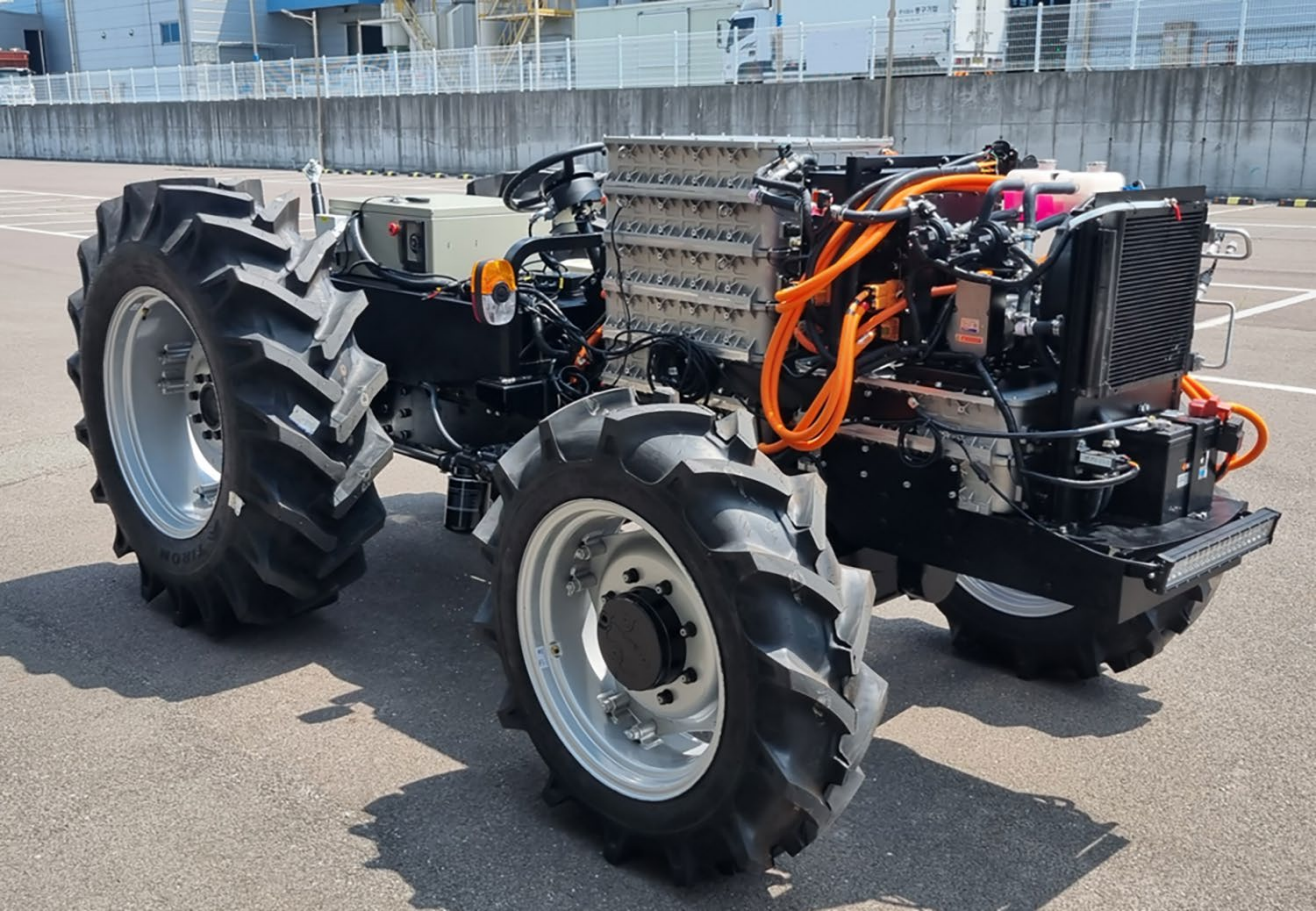
Architecture of the electric tractor platform showing the integration of the electric powertrain, battery system, and structural frame.

**Fig. 2:**
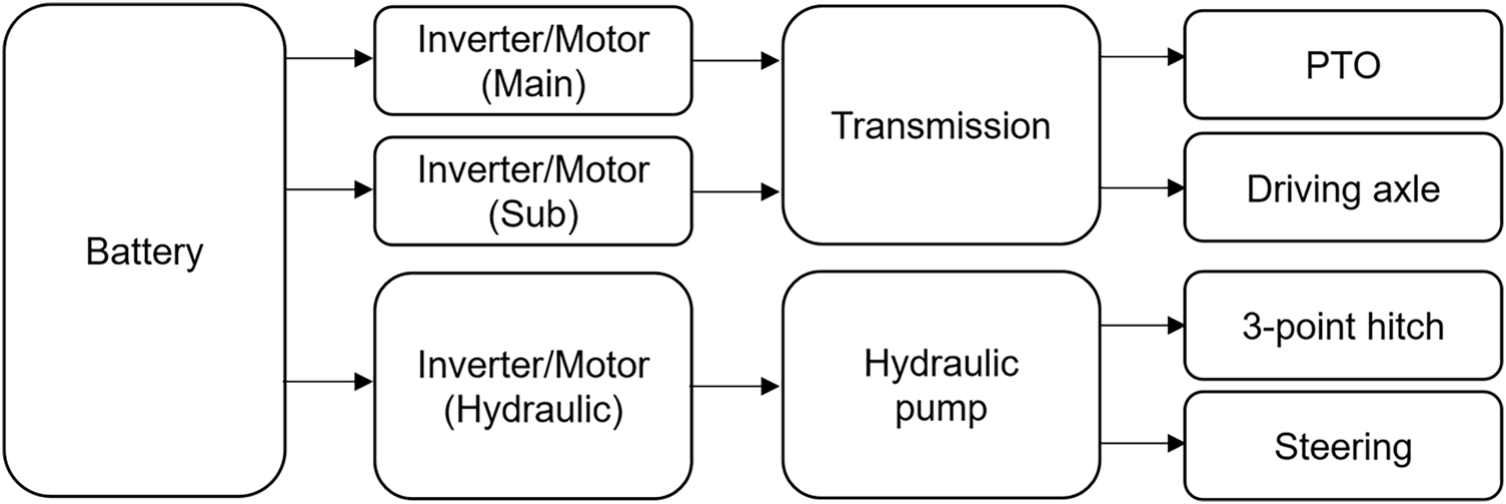
Schematic diagram of the electric tractor platform used in this study.

Figure 1 presents a photograph of the developed electric tractor platform, illustrating the physical implementation of the proposed system architecture, including the integrated battery pack, electric motors, power electronics, and chassis configuration used in this study. The schematic configuration of the electric tractor platform developed in this study is shown in Fig. 2. The proposed platform is a battery-based electric tractor system composed of three electric motors, including a main traction motor, a sub motor for PTO operation, and a dedicated hydraulic motor. The main and sub motors are connected to a shared transmission, through which mechanical power is selectively delivered to the driving axle and the power take-off (PTO), respectively. The PTO motor is rated at 55 kW at 2,200 rpm, delivering a maximum torque of 240 Nm, while the traction motor provides 20 kW at 2,200 rpm with a maximum torque of 87 Nm for vehicle propulsion. In addition, a 20-kW hydraulic motor is independently installed to drive the hydraulic pump. This motor is operated continuously at a speed matched to the rated operating speed of the hydraulic pump, supplying hydraulic power to the three-point hitch and steering system. This configuration enables stable hydraulic performance independent of traction and PTO load conditions. All motors are powered by an 83.1-kWh lithium-ion battery pack, supported by an inverter system and an integrated control unit. The battery capacity allows approximately four hours of continuous field operation under representative agricultural working conditions. The overall system architecture is designed to decouple traction, PTO, and hydraulic power demands, thereby improving operational flexibility and energy management efficiency.

### Structural configuration and characteristics

**Fig. 3:**
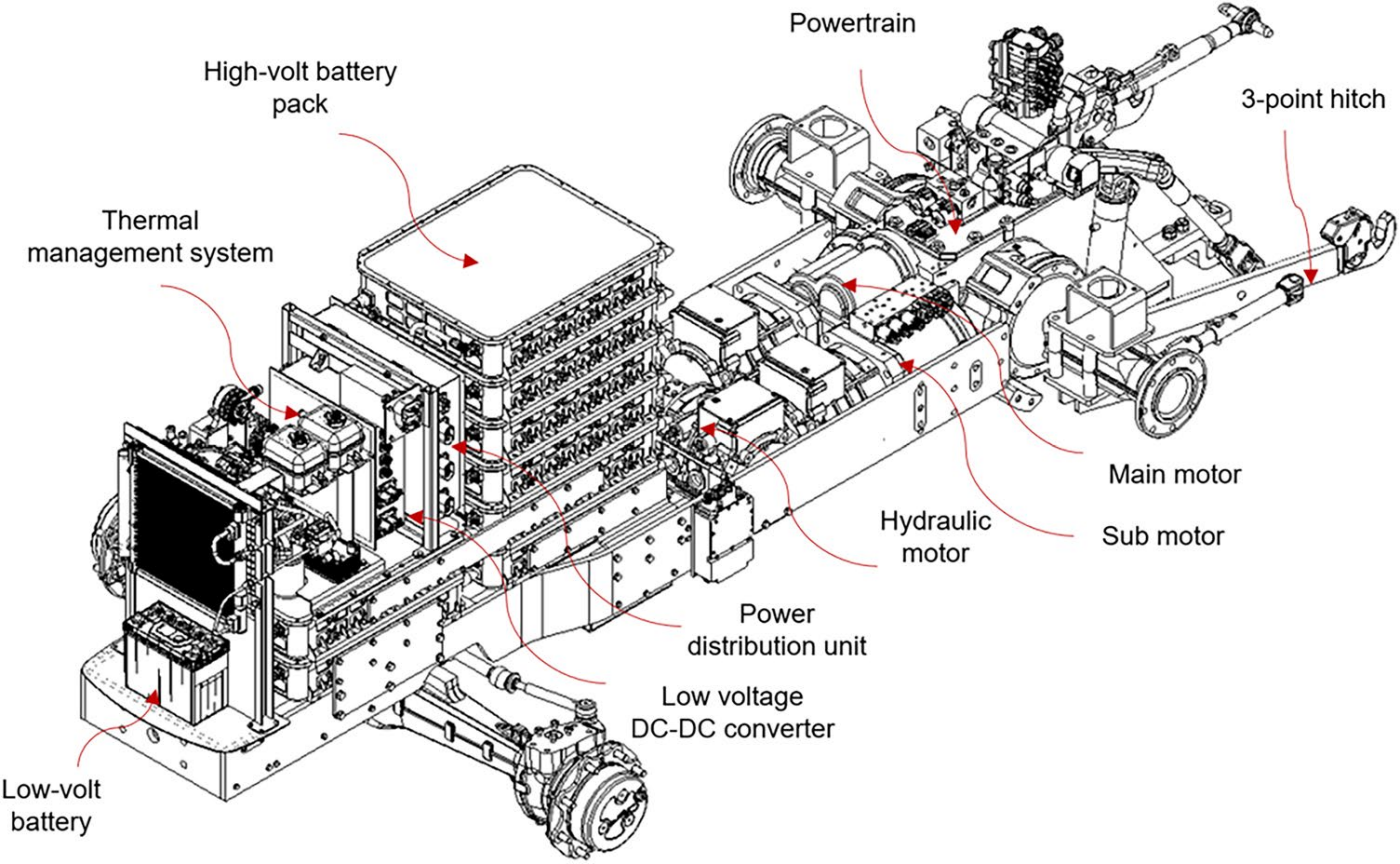
Layout configuration of the electric tractor platform used in this study.

Figure 3 illustrates the layout configuration of the electric tractor platform developed in this study. Unlike conventional agricultural tractors that rely on an engine-centered housing structure, the proposed electric tractor adopts a frame-type platform, onto which the major functional components are modularly mounted. The platform layout is designed based on an engine-replacement concept, in which the conventional internal combustion engine compartment is substituted with a battery-based power system. Accordingly, the high-voltage battery pack is positioned at the location traditionally occupied by the engine, enabling efficient utilization of the available space while maintaining a balanced longitudinal mass distribution.

Key electric powertrain components, including the main motor, sub motor, and reduction gearbox, are arranged along the frame and mechanically connected to the drivetrain and PTO system. A dedicated hydraulic motor is installed to drive the hydraulic pump, supplying hydraulic power to the three-point hitch and steering system. In addition, the 12-V auxiliary battery, along with major charging components and battery-related power electronics, is located at the front region of the platform. This front-oriented packaging strategy facilitates accessibility, thermal management, and electrical integration while contributing to a balanced mass distribution. Based on the overall component arrangement, the platform layout was designed to achieve a front-to-rear axle weight distribution of 51:49, which is commonly regarded as favorable for agricultural tractors in terms of traction performance, steering stability, and operational balance^41^. For comparison, a reference diesel tractor of the same power class exhibits a front-to-rear static load distribution of approximately 42.4:57.6^42^. Although the exact center-of-mass coordinates were not explicitly determined in this study, these axle load distributions provide a representative indication of the longitudinal mass distribution tendency, showing that the electrified platform achieves a more balanced mass layout than a conventional engine-based tractor.

**Fig. 4:**
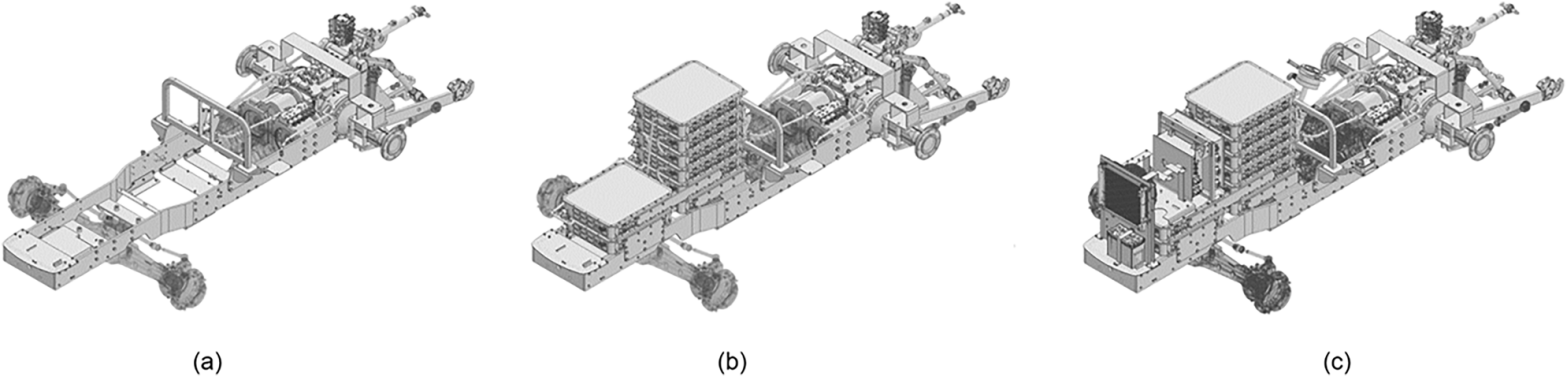
Progressive integration stages and the corresponding mass composition of the electric tractor platform: (**a**) platform, (**b**) battery-integrated platform, and (**c**) fully integrated platform.

Figure 4 illustrates the progressive integration stages of the electric tractor platform, representing three configuration levels: a platform configuration, a battery-integrated configuration, and a fully integrated configuration. These stages were defined to examine how the addition of major subsystems incrementally contributes to the overall mass composition of the electric tractor platform. The platform configuration consists primarily of the structural components, including the modular frame, front axle, and gearbox–rear axle assembly, along with the electric motors and inverters required for basic drivetrain functionality. This configuration represents the baseline platform prior to the integration of energy storage and auxiliary systems. Building upon this baseline, the battery-integrated configuration incorporates the high-voltage battery pack into the platform. This stage captures the mass contribution associated with the integration of the energy storage system while preserving the underlying structural architecture.

The fully integrated configuration further includes auxiliary systems such as the thermal management system, cooling components, wheels and tires, steering system, low-voltage battery, and miscellaneous components required for practical vehicle operation. This configuration represents the complete electric tractor platform as considered in this study. The corresponding component-level mass composition for each integration stage is summarized in Table 1. The total mass increases from 1617 kg for the platform configuration to 2131 kg for the battery-integrated configuration, and to 2900 kg for the fully integrated configuration. These results clearly illustrate the relative mass contributions of the energy storage and auxiliary systems to the overall platform mass. This progressive mass composition provides the basis for evaluating weight distribution and load characteristics, which are subsequently used in the structural performance analyses presented in the following sections.

**Table 1:** Component-level mass composition of the electric tractor platform under different integration stages.

Component category		Platform configuration (kg)	Battery-integrated configuration (kg)	Fully integrated configuration (kg)
Structural components	Frame	253	253	253
	Front axle	350	350	350
	Gearbox and rear axle	800	800	800
Powertrain and energy storage components	Motors and inverters	214	214	214
	High-voltage battery pack	-	514	514
	Low-voltage battery	-	-	10
Auxiliary systems	Thermal management system	-	-	25
	Cooling system	-	-	325
	Wheels and tires	-	-	380
	Steering system	-	-	19
	Miscellaneous components	-	-	10
Total mass		1617	2131	2900

### Simulation analysis

#### Simulation model

**Fig. 5:**
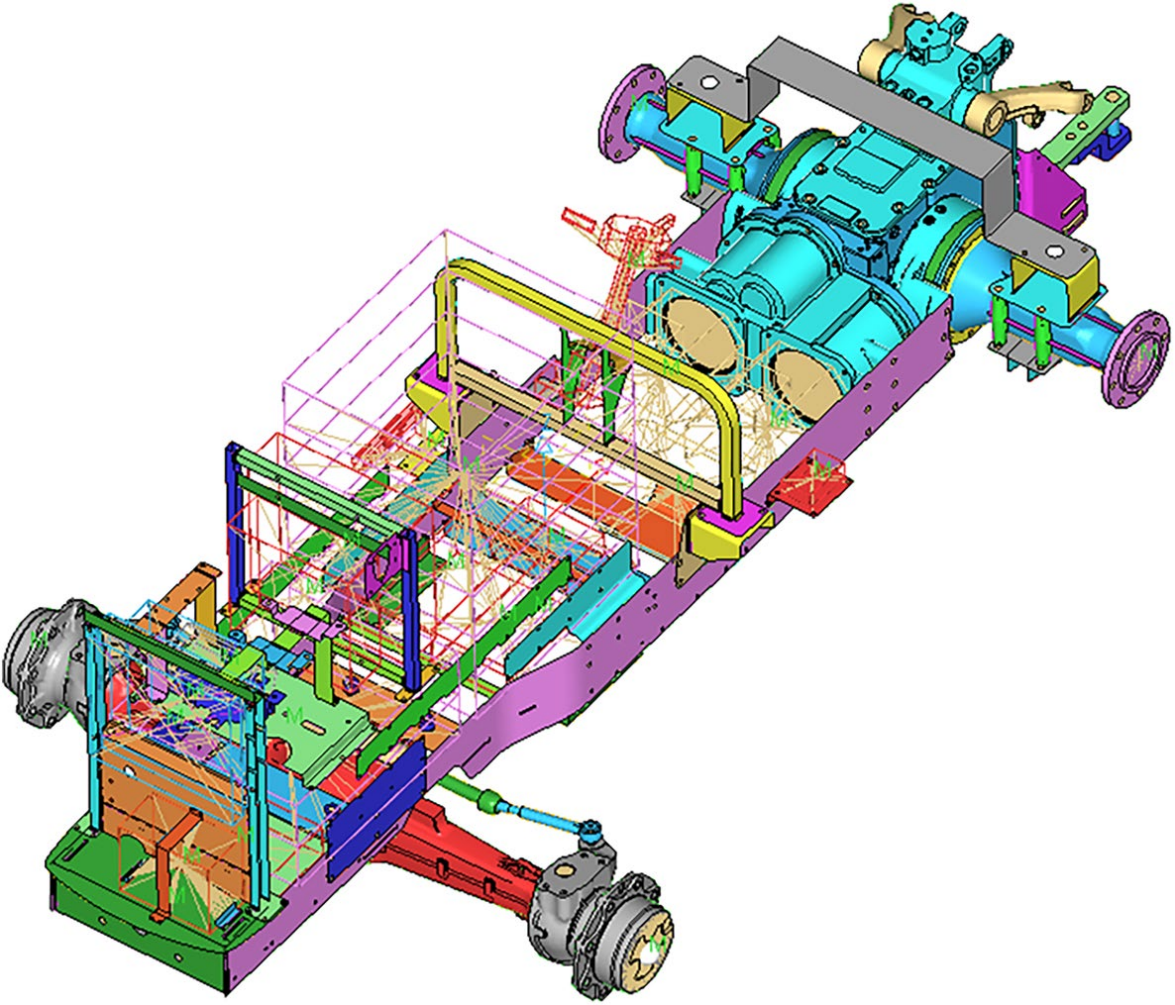
Simplified structural analysis model of the electric tractor platform used for structural strength and free vibration analyses.

Based on the progressive integration stages and mass composition defined in the previous section, numerical simulations were conducted to evaluate the structural characteristics of the electric tractor platform. The simulation framework consisted of (i) structural analysis for strength and stiffness evaluation and (ii) modal analysis for dynamic characteristic assessment. The FE model of the tractor platform was generated using HyperMesh, and all numerical analyses were performed using OptiStruct, a FE solver within the Altair HyperWorks suite (Version 2026, Altair Engineering, USA). The overall FE representation of the electric tractor platform used for the structural and modal analyses is illustrated in Fig. 5. The FE model represents the fully integrated tractor platform including the frame structure and major powertrain components contributing to the overall structural stiffness.

Structural analysis was carried out to investigate the stress distribution and global deformation behavior of the tractor structure under representative loading conditions associated with agricultural operations. The loading conditions were defined based on representative agricultural operating scenarios, including traction loads, vertical loads associated with mounted implements, and braking or impact-related load cases. Appropriate boundary conditions were applied at the axle mounting regions to represent the support constraints of the tractor structure during operation. In this modelling approach, the tire–ground interaction was simplified by applying equivalent support constraints at the axle mounting locations rather than explicitly modelling the tire contact. This method allows the global deformation and load transfer behavior of the tractor frame to be evaluated while maintaining computational efficiency. The calculated stresses were compared with allowable material limits to assess structural safety, while stiffness characteristics were evaluated based on global deformation responses under worst-case loading scenarios.

Modal analysis was performed under free–free boundary conditions to identify the natural frequencies and mode shapes of the tractor structure. The free–free condition was adopted to evaluate the inherent dynamic characteristics of the platform without the influence of external constraints. The dominant vibration modes correspond to typical structural deformation patterns of vehicle frames, including global torsional deformation and vertical and lateral bending modes. These mode shapes provide qualitative insight into the distribution of structural stiffness along the tractor platform and help identify structurally flexible regions of the modular frame architecture. To examine the evolution of dynamic characteristics associated with system integration, modal analyses were conducted using configuration-based structural models corresponding to the platform, battery-integrated, and fully integrated configurations, consistent with the integration stages. These modal characteristics were further analyzed in relation to the structural layout of the tractor platform to interpret the dynamic stiffness behavior of the modular frame.

The FE models were constructed using a combination of 1D beam, 1D rigid, 2D shell, and 3D hexahedral elements selected according to the geometric and mechanical characteristics of each component. Primary load-carrying structural members forming the platform architecture, as well as components connected through welded or bolted joints, were explicitly modeled. In contrast, rubber mounts, spring-type connectors, and auxiliary components with negligible influence on global structural stiffness and strength were excluded to improve computational efficiency and focus the analysis on the primary load-carrying structure^43^. A base mesh size of 5 mm shell elements was adopted for the primary structural components, which was considered sufficient to capture the global structural behavior of the tractor platform while maintaining reasonable computational efficiency^21, 22^. In addition, local mesh refinement was applied in structurally critical regions such as motor mounting interfaces, battery support structures, and frame connection joints where higher stress gradients were expected. The adopted mesh density was verified to provide stable stress and deformation predictions in the primary load-carrying regions of the platform.

Non-structural systems, including the battery pack, thermal management system, electric motors, and electrical components, were incorporated using simplified representations to reflect their mass and stiffness contributions, consistent with the mass composition, while avoiding excessive geometric complexity. The primary load-carrying structural components of the electric tractor platform were made of structural and alloy steels commonly used in agricultural machinery and heavy vehicle structures. The material properties used in the structural strength analysis are summarized in Table 2, based on typical mechanical properties of structural and alloy steels reported in engineering design literature^19, 44^.

**Table 2:** Material properties used in the structural strength analysis of the electric tractor platform.

Component	Material	Young’s modulus (GPa)	Poisson’s ratio	Density (kg/m³)	Yield strength (MPa)
Frame and platform structure	Structural steel	210	0.3	7850	440
Front and rear axles	Alloy steel	210	0.3	7850	600
Mounting structures	Structural steel	210	0.3	7850	355

#### Simulation condition

Structural strength analysis was conducted using the fully integrated electric tractor configuration. To represent realistic driving and working conditions, a total of seven representative load cases were defined, as summarized in Table 3. All loads, deformations, and stiffness directions are defined using a global coordinate system, where the longitudinal, lateral, and vertical directions correspond to the X-, Y-, and Z-axes, respectively. Vertical impact loads associated with vehicle self-weight and road-induced disturbances were represented by severe and moderate vertical impact load cases, corresponding to -4 g and − 2 g accelerations in the vertical direction, respectively. Combined vertical and lateral loading conditions were modeled using front and rear curb impact load cases, in which lateral accelerations of -2 g were applied in combination with vertical accelerations of 1.5 g. Combined vertical and longitudinal loading conditions were represented by front and rear pothole braking load cases, applying longitudinal accelerations of 3 g together with vertical accelerations of 3 g. In addition, a trailing load case was introduced to account for working loads generated by rear-mounted implements, for which a force of 25 kN was applied at the hitch point^41^. All loading cases were combined with gravitational loading to reflect realistic operating conditions. Structural strength was evaluated based on the maximum von Mises stress obtained under each load case, and the calculated stresses were compared with the material strength limits of the frame to assess structural safety.

**Table 3:** Summary of loading conditions applied in the structural strength analysis.

Load case		X-axis (Longitudinal)	Y-axis (Lateral)	Z-axis (Vertical)
1	Severe vertical impact load	-	-	-4 g
2	Moderate vertical impact load	-	-	-2 g
3	Front curb impact load	-	-2 g	1.5 g
4	Front pothole braking load	3 g	-	3 g
5	Rear curb impact load	-	-2 g	1.5 g
6	Rear pothole braking load	3 g	-	3 g
7	Trailing load	25 kN	-	-

*Acceleration loads are expressed in g, while the trailing load is applied as an equivalent force (kN).

Following the strength evaluation, static stiffness analysis was performed to assess the global deformation behavior of the electric tractor platform. Representative loading and boundary conditions were defined to evaluate the torsional and bending stiffness characteristics of the structure. Torsional stiffness was assessed by constraining one axle and applying a torsional load to the opposite axle, while the resulting angular deformation of the frame was measured. Bending stiffness was evaluated by constraining both axles and applying a vertical load to the central region of the frame, with the resulting vertical displacement used to quantify the global bending response.

In addition to the static structural analyses, modal analysis was conducted to identify the inherent dynamic characteristics of the electric tractor platform. The analysis was performed under free–free boundary conditions to exclude the influence of external constraints. To examine the effect of progressive system integration on dynamic behavior, modal analyses were carried out for the platform, battery-integrated, and fully integrated configurations, consistent with the integration stages defined earlier. The natural frequencies and corresponding mode shapes were extracted for each configuration, with particular emphasis on low-frequency modes relevant to operational vibration behavior.

### Methodology

**Fig. 6:**
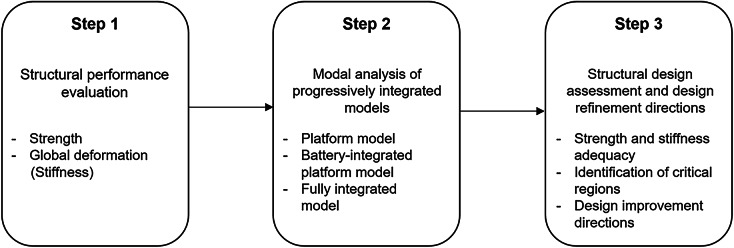
Flowchart of the structural analysis and evaluation procedure for the electric tractor platform.

The overall evaluation procedure adopted in this study is summarized as in Fig. 6. First, the structural performance of the fully integrated electric tractor model was evaluated through strength and stiffness analysis to assess structural safety under representative worst-case loading conditions. Second, modal analysis of configuration-based progressively integrated models was conducted to investigate changes in global dynamic characteristics arising from battery and system integration. Finally, the results of structural and modal analyses were synthesized to derive structural analysis conclusions and design improvement insights, identifying critical regions and potential directions for future structural refinement.

To quantitatively evaluate the structural performance in each analysis step, representative mechanical indices were defined based on the FE analysis results. Structural strength was assessed using the von Mises equivalent stress, as defined in Eq. (1), which provides a unified measure of multiaxial stress states. Structural safety was evaluated by comparing the calculated maximum von Mises stress with the material strength limit, as expressed in Eq. (2). This stress-based criterion was used to identify critical load cases and structurally vulnerable regions under severe operating conditions.

1 $$\:{\sigma\:}_{vm}=\sqrt{\frac{1}{2}\left[{\left({\sigma\:}_{1}-{\sigma\:}_{2}\right)}^{2}+{\left({\sigma\:}_{2}-{\sigma\:}_{3}\right)}^{2}+{\left({\sigma\:}_{3}-{\sigma\:}_{1}\right)}^{2}\right]},$$

2 $$\:{\sigma\:}_{vm}\le\:{\sigma\:}_{ult},$$

where $\:{{\sigma\:}_{vm}}_{F}$ is von Mises equivalent stress (MPa), $\:{\sigma\:}_{1}$, $\:{\sigma\:}_{2}$, $\:{\sigma\:}_{3}$ are the principal stresses (MPa), $\:{\sigma\:}_{ult}$ is the ultimate tensile strength of the frame material (MPa).

Static stiffness characteristics were evaluated in terms of torsional stiffness and bending stiffness, which were calculated using Eq. (3) and Eq. (4), respectively. These stiffness indices were selected to represent the global rigidity of the tractor frame, as torsional stiffness is closely related to vehicle handling stability, while bending stiffness reflects load-carrying capability and resistance to vertical deflection. The defined stiffness metrics enable consistent comparison of structural rigidity under different configuration conditions.

3 $$\:{K}_{t}=\frac{T}{\theta\:},$$

4 $$\:{K}_{b}=\frac{F}{\delta\:},$$

where $\:{K}_{t}$ is the torsional stiffness (kNm/deg), $\:T$ is applied torque (Nm), $\:\theta\:$ is the resulting twist angle (deg), $\:{K}_{b}$ is the bending stiffness (kN/mm), $\:F$ is the applied vertical load (Nm), $\:\delta\:$ is the vertical displacement (mm).

The global dynamic characteristics of the tractor frame were evaluated through modal analysis. The relationship between natural frequency, structural stiffness, and mass was conceptually described using Eq. (5). Although the natural frequency is not a direct measure of stiffness, this relationship indicates that modal characteristics reflect the combined effects of structural stiffness and mass. Accordingly, Eq. (5) was used to interpret variations in modal characteristics resulting from system integration and to support comparative discussion between the electric tractor and a conventional tractor of the same power class. Based on these formulations, the structural strength, static stiffness, and modal characteristics were systematically evaluated using Eqs. (1)–(5), which form the quantitative basis for the results and discussions presented in this study.

5 $$\:{f}_{n}=\frac{1}{2\pi\:}\sqrt{\frac{k}{m}},$$

where $\:{f}_{n}$ is the natural frequency (Hz), $\:k$ is equivalent structural stiffness (N/m), $\:m$ is the equivalent mass of the system (kg).

## Results

### Structural analysis results

The structural strength analysis results of the fully integrated electric tractor platform under representative loading conditions are summarized in Table 4, while a quantitative comparison of the maximum stress levels for each load case is provided in Table 5. The evaluation focused on identifying critical loading scenarios and structurally vulnerable regions by comparing the maximum von Mises stress with the yield strength of the structural materials. The yield strengths of the structural materials used in the analysis range from 355 MPa to 600 MPa depending on the component type. This comparison enables the identification of load cases where the stress level approaches or exceeds the material strength limits, thereby providing insights into potential structural reinforcement requirements and safety margin considerations in the platform design. Although several severe loading scenarios resulted in stress levels exceeding the nominal yield strength, these cases represent extreme impact conditions intended to evaluate the structural robustness and identify potential design improvements. The comparison therefore serves as a conservative assessment of structural safety and safety margin under representative agricultural operating conditions.

**Table 4:** von Mises stress distributions of the fully integrated electric tractor platform under representative structural strength load cases.

Load case	Global stress distribution	Local stress distribution
1		
2		
3		
4		`
5		
6		
7		

**Table 5:** Comparison of maximum von Mises stress values for different structural strength load cases.

Load case		Max. stress (MPa)
1	Severe vertical impact load	1019
2	Moderate vertical impact load	520
3	Front curb impact load	311
4	Front pothole braking load	849
5	Rear curb impact load	220
6	Rear pothole braking load	542
7	Trailing load	47

Under the severe vertical impact load, the maximum von Mises stress reached 1019 MPa, which significantly exceeded the nominal yield strength of the platform structural steel. Similarly, the moderate vertical impact load resulted in a maximum stress of 520 MPa, also exceeding the allowable range. In both cases, elevated stress levels were primarily observed in the cross-member region, indicating a strong sensitivity of this component to vertical impact loading conditions. For the front curb impact load, the maximum stress was 311 MPa, which remained below the material strength limit, indicating stable structural behavior under moderate lateral impact loading. In contrast, the front pothole braking load produced a substantially higher maximum stress of 849 MPa, exceeding the material strength limit. This result highlights the pronounced influence of combined longitudinal braking and vertical excitation on the global stress response of the tractor frame. In the rear curb impact load case, the maximum stress was 220 MPa, well within the allowable strength range, demonstrating sufficient structural safety under rear lateral impact loading. Conversely, the rear pothole braking load resulted in a maximum stress of 542 MPa, again exceeding the material strength limit, confirming that braking events combined with vertical excitation constitute one of the most demanding loading scenarios for the tractor frame. Under the trailing load condition, which represents typical working loads transmitted through rear-mounted implements, the maximum stress was only 47 MPa, indicating a substantial margin of safety relative to the material strength limit. This result suggests that the tractor frame experiences comparatively low stress levels under steady operational working loads.

Overall, the structural strength analysis demonstrates clear differences in stress response depending on the applied loading conditions. While several load cases remained within the material strength limit, impact- and braking-dominated loading scenarios consistently resulted in higher stress levels providing a basis for comparative evaluation of critical operating conditions. These extreme loading scenarios were intentionally considered to identify structurally vulnerable regions and evaluate the robustness of the platform design. Since the analyzed platform represents a prototype electric tractor configuration, the structural analysis also provides guidance for future design refinement. The identified stress concentrations highlight structural regions that may benefit from reinforcement in subsequent prototype iterations.

**Fig. 7:**
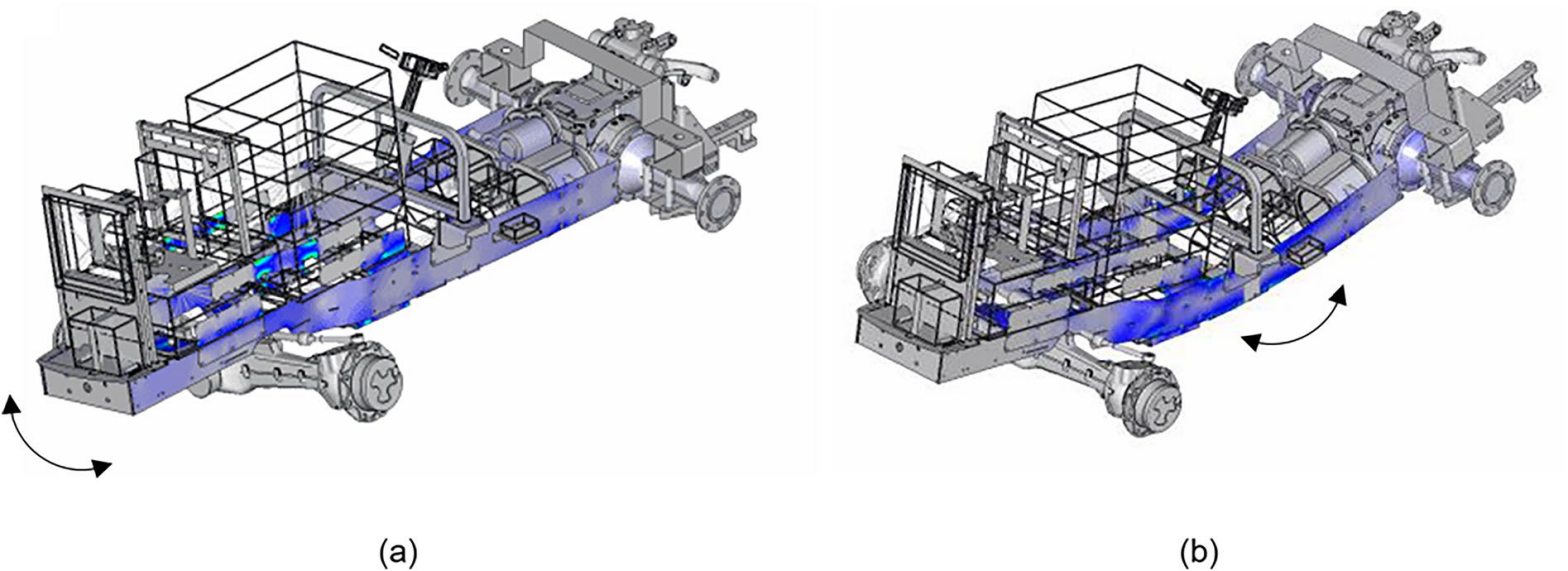
Global deformation patterns of the fully integrated electric tractor platform: (**a**) torsional and (**b**) bending loading conditions.

The static stiffness characteristics of the fully integrated electric tractor platform were evaluated in terms of torsional and bending stiffness. The global deformation patterns obtained under torsional and bending loading conditions are illustrated in Fig. 7. The torsional stiffness of the integrated frame was calculated as 9.5 kNm/deg, while the bending stiffness was evaluated as 17.9 kN/mm. These stiffness values characterize the global structural rigidity and deformation behavior of the integrated tractor frame. Compared with the platform-level configuration, the integration of major system components contributed to an overall increase in global bending stiffness, indicating enhanced load-carrying capability of the frame. A quantitative summary of the evaluated static stiffness values is provided in Table 6. Similar structural evaluation approaches based on FE analysis have been widely applied in the design and assessment of off-road vehicle chassis to examine structural rigidity and deformation behavior under representative loading conditions^28, 45^.

**Table 6:** Summary of static stiffness values and associated dynamic characteristics of the fully integrated electric tractor platform.

Static stiffness		Dynamic characteristics		
Bending (kN/mm)	Torsion (kNm/deg)	Torsion (Hz)	Vertical bending (Hz)	Lateral bending (Hz)
17.9	9.5	8.5	26.5	9.1

The spatial distribution of stiffness revealed non-uniform structural characteristics across the frame. Regions associated with the front axle mounting exhibited relatively stable stiffness behavior due to the presence of the axle assembly and associated structural constraints. In contrast, regions characterized by abrupt changes in frame width showed comparatively lower stiffness levels, indicating localized compliance within the frame structure. The central region of the frame, where cross members are sparsely arranged, exhibited relatively lower stiffness compared with other regions, whereas the rear axle mounting region demonstrated high stiffness due to the rigid connection between the frame and rear axle assembly.

In addition to the static stiffness results, dynamic characteristics of the tractor platform were evaluated using the natural frequencies obtained from modal analysis. The first torsional mode was identified at 8.5 Hz, while the vertical and lateral bending modes occurred at 26.5 Hz and 9.1 Hz, respectively. Although natural frequency is not a direct measure of stiffness, it provides complementary insight into the combined effects of structural stiffness and mass distribution of the tractor frame. The torsional stiffness was evaluated by applying opposing vertical loads at the axle locations to induce frame twisting, while the bending stiffness was obtained from the vertical deformation response under static loading conditions.

### Modal analysis results

**Fig. 8:**
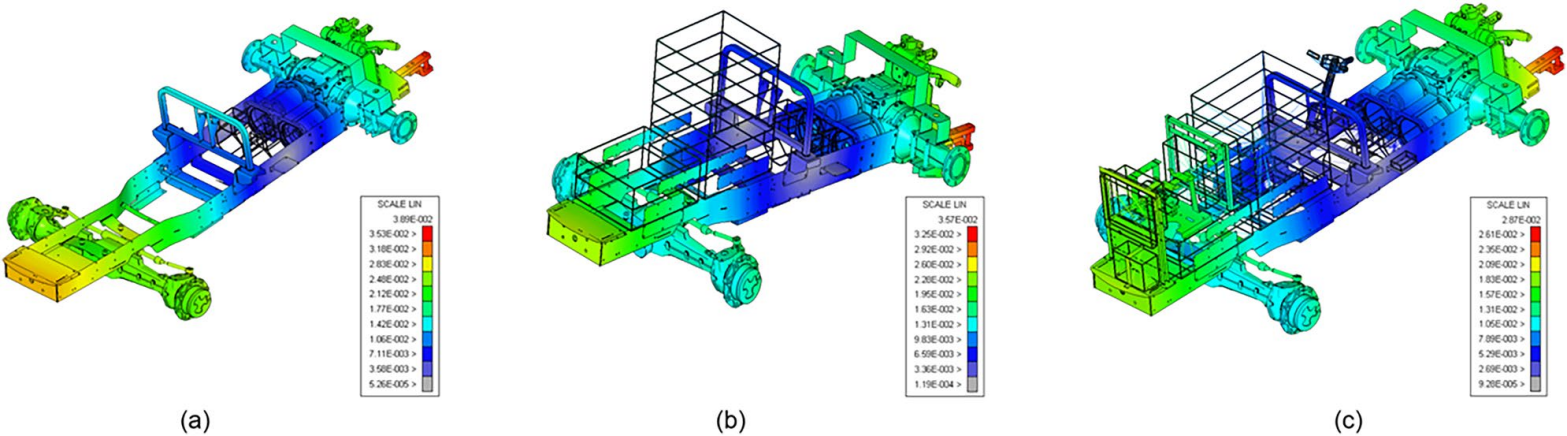
Representative deformation patterns obtained from free–free modal analysis of the electric tractor platform under different system integration stages: (**a**) platform, (**b**) battery-integrated platform, and (**c**) fully integrated platform. Overall, the modal analysis results demonstrate that progressive system integration significantly alters the dynamic behavior of the electric tractor platform. While global vibration modes remain within the low-frequency range relevant to vehicle operation, their frequency shifts reflect changes in mass distribution and structural stiffness. The comparison across integration stages confirms that the dynamic characteristics of the tractor frame are governed by the balance between stiffness enhancement and mass addition introduced through system integration.

Free–free modal analysis was performed to investigate the evolution of global and local dynamic characteristics of the electric tractor platform with progressive system integration. Representative deformation patterns for each integration stage are illustrated in Fig. 8, and the corresponding natural frequencies and vibration mode classifications are summarized in Table 7.

**Table 7:** Comparison of natural frequencies and corresponding vibration modes of the electric tractor platform under different system integration stages.

Mode	Platform (Hz/ mode)		Battery-integrated platform (Hz/ mode)		Fully integrated platform (Hz/ mode)	
1st	4.4	Front axle bounce mode	4.6	Front axle bounce mode	4.2	Front axle bounce
2nd	6.7	Global lateral bending mode*	8.5	Global lateral bending mode*	6.5	Local structural mode (Auxiliary system-dominated)
3rd	8.4	Global torsional mode*	12.3	Global torsional mode*	8.5	Global torsional mode*
4th	27.1	Local structural mode	25.0	Global lateral bending mode*	9.1	Global lateral bending mode*
5th	29.3	Local structural mode	27.1	Local structural mode	15.2	Local structural mode
6th	29.5	Local structural mode	28.5	Local structural mode*	15.7	Local structural mode
7th	30.8	Local structural mode	29.4	Local structural mode	15.9	Local structural mode
8th	32.3	Local structural mode	31.3	Local structural mode	16.8	Local structural mode
9th	33.5	Local structural mode	33.7	Local structural mode	18.9	Local structural mode
10th	34.4	Local structural mode	34.3	Local structural mode	23.9	Local structural mode

*Dominant global vibration modes involving coherent deformation of the main frame structure and governing the overall dynamic behavior of the tractor frame.

For the platform model, the dominant low-frequency modes consisted of a front axle bounce mode at 4.4 Hz, followed by global lateral bending and global torsional modes at 6.7 Hz and 8.4 Hz, respectively. These modes indicate limited global stiffness of the bare frame structure, particularly in torsional and lateral bending directions, making the platform more susceptible to global deformation under dynamic excitation. With integration of the battery pack, a general increase in natural frequencies was observed in the dominant global modes. The global lateral bending mode shifted from 6.7 Hz to 8.5 Hz, while the global torsional mode increased from 8.4 Hz to 12.3 Hz. This trend suggests that the battery pack contributes not only additional mass but also enhanced load-sharing and structural reinforcement effects, leading to improved global stiffness characteristics. However, the presence of multiple local structural modes at higher frequencies indicates that stiffness enhancement is not uniformly distributed across the entire structure. In the fully integrated platform model, the dominant global modes were identified as the global torsional mode at 8.5 Hz and the global lateral bending mode at 9.1 Hz, following the front axle bounce mode at 4.2 Hz. Compared with the battery-integrated configuration, the torsional mode frequency decreased due to additional mass contributions from cooling systems, while the lateral bending mode remained within a similar frequency range. This behavior reflects the combined effects of mass increase and stiffness enhancement associated with full system integration. Higher-order modes above approximately 15 Hz were consistently classified as local structural modes, indicating that they are governed by localized component deformation rather than coherent global frame motion.

### Structural design assessment

**Fig. 9:**
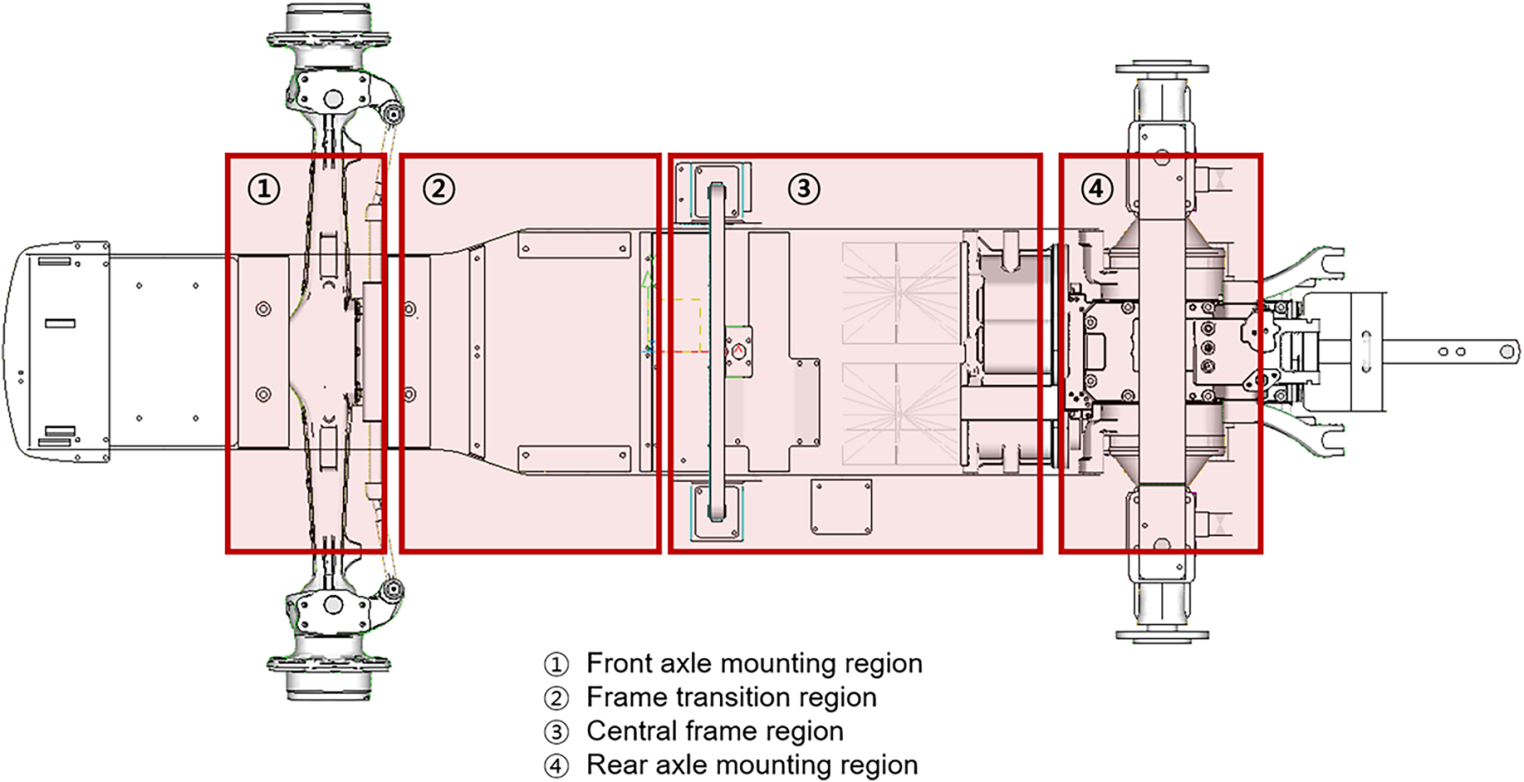
Region-based segmentation of the electric tractor platform for structural design assessment highlighting four representative regions considered in the strength, stiffness, and modal analyses.

Structural design assessment was conducted by synthesizing the results of structural strength, static stiffness, and free vibration analyses performed on the fully integrated electric tractor platform under representative operating and loading conditions. These analyses include the structural strength evaluation under impact and braking load cases, the global stiffness characteristics of the frame structure, and the dynamic behavior identified through modal analysis. To support a systematic interpretation of the structural response, the frame was examined in terms of four representative structural regions, as illustrated in Fig. 9, each contributing differently to the overall structural performance of the platform.

Region ①, corresponding to the front axle mounting area, exhibited stable structural behavior across all analyses due to the rigid integration of the axle assembly and the effective load transfer path between the axle and the frame structure. This region primarily governs the first global vibration mode associated with front axle bounce while maintaining relatively low stress concentration. Region ②, associated with geometric transitions in frame width, showed comparatively reduced stiffness and increased participation in global bending deformation, indicating higher structural compliance under combined loading conditions. Region ③, representing the central frame portion, was identified as the structurally critical region of the platform. This region exhibited pronounced torsional deformation and localized stress concentration under impact- and braking-dominated load cases. The observed behavior is consistent with the global deformation characteristics identified in the modal analysis results, where torsional and lateral bending modes play a dominant role in the dynamic response of the frame structure. These results indicate that the central frame region significantly influences the global load transfer behavior of the integrated tractor platform. In contrast, Region ④, corresponding to the rear axle mounting area, demonstrated the highest structural stability due to the rigid connection between the rear axle assembly and the surrounding frame structure, resulting in minimal deformation and limited participation in dominant global vibration modes.

Overall, the region-based interpretation demonstrates that the structural performance of the electric tractor platform is governed not only by global stiffness levels but also by the spatial distribution of structural members and load paths. In particular, the central frame region was identified as a key area influencing both strength and dynamic behavior, suggesting that targeted structural reinforcement or geometric optimization in this region would be the most effective strategy for improving overall durability and robustness without excessive increase in structural mass. Unlike conventional diesel tractors, where the engine block directly contributes to the primary load-bearing path of the chassis, the proposed electrified platform redistributes structural loads through modular frame members and integrated structural components. As a result, the central frame region plays a more significant role in transferring loads between the front and rear axle mounting structures, making careful structural design of this region essential for maintaining global structural integrity. Although detailed fatigue life prediction was beyond the scope of the present study, the identified structural response characteristics provide useful insight for future durability-oriented design and fatigue assessment of electrified tractor platforms.

In this section, the structural performance of the developed tractor platform is evaluated through comparison with a reference tractor of the same power class. The reference tractor is a commercially available diesel-engine tractor employing a conventional engine–axle integrated structural architecture, in which the engine and drivetrain components contribute to the global load-bearing capability of the platform. As such, it represents the structural performance level typically required for tractors within this power rating and provides a consistent baseline for assessing the adequacy of the developed platform^12^. To ensure a fair comparison, both the reference and developed models represent fully assembled tractor platforms and were analyzed under identical loading and boundary conditions.

**Fig. 10:**
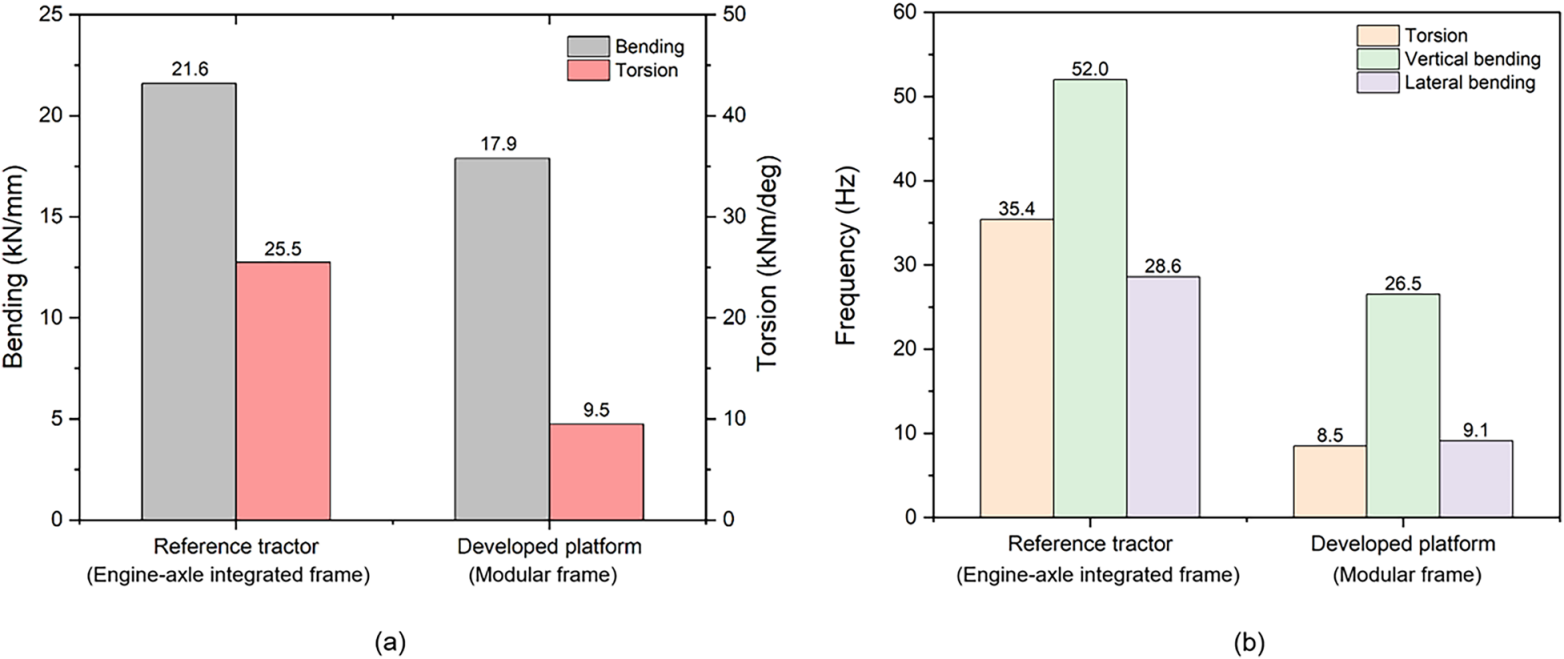
Comparison of structural performance between the proposed platform and a reference tractor of the same power class: (**a**) static stiffness in bending and torsion, and (**b**) dynamic characteristics based on dominant natural frequencies.

Figure 10 presents a comparative assessment of the static stiffness and dynamic characteristics of the developed platform relative to the reference tractor platform. In terms of static performance, the developed platform exhibits bending and torsional stiffness values of 17.9 kN/mm and 9.5 kNm/deg, respectively, compared to 21.6 kN/mm and 25.5 kNm/deg for the reference diesel tractor. Although the stiffness values of the developed platform are lower than those of the reference platform, they remain within an acceptable structural range for tractors of the same power class. This indicates that the modular electrified architecture can maintain global structural integrity despite the absence of the load-bearing engine block found in conventional engine–axle integrated structures. In contrast to conventional diesel tractors where the engine block contributes to the primary load-bearing path, the proposed electrified platform redistributes structural loads through the frame members and integrated structural components.

The dynamic characteristics indicate that the developed platform exhibits lower dominant natural frequencies than the reference tractor across the global torsional, vertical bending, and lateral bending modes. Specifically, the torsional mode frequency decreases from 35.4 Hz to 8.5 Hz, while the vertical and lateral bending modes decrease from 52.0 Hz to 28.6 Hz to 26.5 Hz and 9.1 Hz, respectively. This reduction in natural frequencies is primarily attributed to the increased system mass associated with the integration of electrification-related components, together with changes in structural stiffness distribution resulting from the modular platform architecture. Nevertheless, the dominant global vibration modes of the developed platform remain sufficiently separated from typical excitation sources encountered during agricultural operation.

## Conclusions

This study evaluated the structural performance of an electrified tractor platform through integrated analyses of structural strength, static stiffness, and free vibration characteristics. A fully integrated tractor platform model was developed to investigate the influence of electrification-related mass distribution and component integration on both global and local structural behavior. The structural strength analyses under representative impact, braking, and implement-induced loading conditions confirmed that the electrified tractor platform satisfies structural safety requirements for typical agricultural operating scenarios. Localized stress concentrations were observed under several impact- and braking-dominated load cases; however, these were associated with localized load transfer paths rather than limitations in the overall structural stiffness of the frame. The static stiffness evaluation demonstrated that the fully integrated electric tractor platform provides sufficient global bending and torsional stiffness. Despite the absence of a conventional engine block acting as a primary structural member, the modular frame structure maintained stiffness levels adequate to ensure steering stability and to suppress excessive structural deformation under typical operating conditions. Modal analysis indicated that system integration leads to reduced dominant natural frequencies due to the increased structural mass associated with electrification. Nevertheless, the primary global vibration modes remain sufficiently separated from typical excitation sources encountered during agricultural operations. A comparative assessment with a conventional diesel tractor platform employing an engine–axle integrated structural architecture further confirmed that, while electrification-related mass integration slightly lowers the natural frequencies, the overall static stiffness and dynamic characteristics of the proposed modular platform remain structurally appropriate. Overall, the results demonstrate the structural feasibility of modular electrified tractor platform architectures and clarify the influence of electrification-induced architectural changes on vehicle structural behavior. The findings provide a structural design basis for the development of electrified agricultural machinery platforms. Future work will focus on experimental validation using a full-scale prototype in order to correlate numerical predictions with measured structural and dynamic responses under realistic agricultural operating conditions.

## Data Availability

The datasets used and analyzed during the current study available from the corresponding author, [Y.J.K.], on reasonable request.

## References

[1] Do, Y. W. et al. Determining exhaust emissions (CO, NOx, PM) for a combine harvester based on measured engine load and emission factors using PEMS during actual field operation. *Comput. Electron. Agric.* **231**, 110026 (2025).

[2] Kim, W. S. et al. Evaluation of exhaust emissions of agricultural tractors using portable emissions measurement system in Korean paddy field. *Sci. Rep.* **14**, 1–14 (2024). 38347145 10.1038/s41598-024-53995-0 PMC11303376

[3] Olkkonen, V., Lind, A., Rosenberg, E. & Kvalbein, L. Electrification of the agricultural sector in Norway in an effort to phase out fossil fuel consumption. *Energy* **276**, 127543 (2023).

[4] Gong, S. Y. et al. Prediction of tractor drawbar pull under different tillage tools using machine learning and low-cost sensors. *Sci Rep* **15** (2025). 10.1038/s41598-025-24974-w PMC12635318 41266736

[5] Deng, X. et al. Research on dynamic analysis and experimental study of the distributed drive electric tractor. *Agriculture* **13** (2023).

[6] Siddique, M. A. A. et al. Effect of motor speeds on traction performance of a single-motor electric tractor at various gear stages during plow tillage. *Sci. Rep.* **15**, 1–12 (2025). 40695907 10.1038/s41598-025-04913-5 PMC12284163

[7] Mao, Y., Wu, Y., Yan, X., Liu, M. & Xu, L. Simulation and experimental research of electric tractor drive system based on Modelica. *PLoS One*. **17**, 1–20 (2022). 10.1371/journal.pone.0276231 PMC9671462 36395258

[8] Zhang, J. et al. Design and optimization of dual-motor electric tractor drive system based on driving cycles. *PLoS One*. **18**, 1–22 (2023). 10.1371/journal.pone.0286378 PMC10237445 37267273

[9] Tong, Y., Zhang, J., Xu, L. & Yan, X. Driving System Design and Power Source Parameter Optimization of Tractor with Dual-Motor Coupling Drive. *World Electr. Veh. J* **14** (2023).

[10] Melo, R. R. et al. Conception of an electric propulsion system for a 9 kW electric tractor suitable for family farming. *IET Electric Power Appl.*. 10.1049/iet-epa.2019.0353 (2019).

[11] Kim, N., Yan, Z., Vijayagopal, R., Jung, J. & He, X. Evaluation of Advanced Powertrain Technologies for Large Agricultural Tractors: Insights on Energy Consumption and Life Cycle Environmental Impact. *SAE Int. J. Sustain. Transp. Energy Environ. Policy*. **06**, 221–242 (2025).

[12] Baek, S. M., Jeon, H. H., Kim, W. S., Kim, Y. S. & Kim, Y. J. Design and analysis of a power transmission system for 55 kW electric tractor using agricultural workload data. *Sci. Rep.* **15**, 1–17 (2025). 40745442 10.1038/s41598-025-11444-6 PMC12313879

[13] Dizo, J. et al. Static analysis of single-axle tractor trialer frame. *Eng. Rural Dev.* **20**, 534–541 (2021).

[14] Kim, J. H., Gim, D. H. & Nam, J. S. Experimental structural safety analysis of front-end loader of agricultural tractor. *Agriculture* **14** (2024).

[15] Yoo, H. G. et al. Effect of hybrid metal-composite gear on the reduction of dynamic transmission error. *J. Mech. Sci. Technol.* **37**, 3445–3457 (2023).

[16] Wang, Z., Zhou, J., Wang, X. & Sunusi, I. I. Design and verification of a modular reconfigurable test platform for electric tractors. *Appl. Sci.* **11**, 1–18 (2021).

[17] Wen, C. et al. Power density based fatigue load spectrum editing for accelerated durability testing for tractor front axles. *Biosyst Eng.* **200**, 73–88 (2020).

[18] Kim, J. H. et al. Optimization of accelerated life test design process for gears and bearings using equivalent damage method. *Sci. Rep.* **15**, 1–14 (2025). 41107369 10.1038/s41598-025-20451-6 PMC12534414

[19] Zhang, X. et al. The anti-fatigue lightweight design of heavy tractor frame based on a modified decision method. *Struct. Multidiscip Optim.* **65**, 1–17 (2022).

[20] Dong, S., Li, S., Fu, S. & Wang, K. Finite element analysis and optimization of tractor gearbox body under various kinds of working conditions. *Sci. Rep.* **12**, 1–17 (2022). 36253397 10.1038/s41598-022-22342-6 PMC9576754

[21] Hruban, V., Drobitko, A., Khramov, M. & Tovpyha, M. Strength analysis and optimisation of trailer agricultural machinery structures using finite element methods. *Mach. Energ.* **16**, 117–130 (2025).

[22] Cascino, A., Meli, E. & Rindi, A. High-Fidelity Finite Element Modelling (FEM) and Dynamic Analysis of a Hybrid Aluminium–Honeycomb Railway Vehicle Carbody. *Appl. Sci.* **16** (2026).

[23] Luo, R. K., Gabbitas, B. L. & Brickle, B. V. Fatigue life evaluation of a railway vehicle bogie using an integrated dynamic simulation. *Proc. Inst. Mech. Eng. Part F J. Rail Rapid Transit* **208**, 123–132 (1994).

[24] Cascino, A., Meli, E. & Rindi, A. Design and Optimization of a Hybrid Railcar Structure with Multilayer Composite Panels. *Mater. (Basel)*. **18**, 1–15 (2025). 10.3390/ma18215013 PMC12609127 41227971

[25] Tang, J. et al. Laminate design, optimization, and testing of an innovative carbon fiber-reinforced composite sandwich panel for high-speed train. *Polym. Compos.* **42**, 5811–5829 (2021).

[26] Berardi, A. et al. FEA for Optimizing Design and Fabrication of Frame Structure of Elevating Work Platforms. *Appl. Sci.* **15**, 1–15 (2025).

[27] Benmeddah, A. et al. Modeling and Experimental Validation of an Off-Road Truck’s (4 × 4) Lateral Dynamics Using a Multi-Body Simulation. *Appl. Sci.* **14** (2024).

[28] Kumar Dubey, K., Pathak, B., Singh, K., Rathore, B. P. & Raghav Singh Yadav, S. Mechanical strength study of Off-Road vehicle chassis body materials. *Mater. Today Proc.* **46**, 6682–6687 (2020).

[29] Yang, P., Zang, M., Zeng, H. & Guo, X. The interactions between an off-road tire and granular terrain: GPU-based DEM-FEM simulation and experimental validation. *Int J. Mech. Sci.* **179** (2020).

[30] Wang, L. et al. Advances in tractor rollover and stability control: Implications for off-road driving safety. *Comput. Electron. Agric.* **226**, 109483 (2024).

[31] Cascino, A., Meli, E., Rindi, A., Pucci, E. & Matoni, E. Experimental Validation and Dynamic Analysis of Additive Manufacturing Burner for Gas Turbine Applications. *Machines* **13**, 1–18 (2025).

[32] Hu, M., Zeng, J., Xu, S., Fu, C. & Qin, D. Efficiency Study of a Dual-Motor Coupling EV Powertrain. *IEEE Trans. Veh. Technol.* **64**, 2252–2260 (2015).

[33] Xie, B. et al. Design and hardware-in-the-loop test of a coupled drive system for electric tractor. *Biosyst Eng.* **216**, 165–185 (2022).

[34] Kim, N. et al. Development of the Power- and Usage-Based Simulator for Evaluating Off-Road Mobile Machinery Energy Consumption. *SAE Tech. Pap*. 1–11. 10.4271/2025-01-8595 (2025).

[35] De Carlo, M. & Mantriota, G. Electric vehicles with two motors combined via planetary gear train. *Mech. Mach. Theory* **148** (2020).

[36] Linares, P., Méndez, V. & Catalán, H. Design parameters for continuously variable power-split transmissions using planetaries with 3 active shafts. *J. Terramechanics*. **47**, 323–335 (2010).

[37] Mantriota, G. & Reina, G. Dual-motor planetary transmission to improve efficiency in electric vehicles†. *Machines* **9**, 1–17 (2021).

[38] Saiteja, P., Ashok, B. & Upadhyay, D. Evaluation of electric vehicle performance characteristics for adaptive supervisory self-learning-based SR motor energy management controller under real-time driving conditions. *Vehicles* **6**, 509–538 (2024).

[39] Miranda, M. H. R., Silva, F. L., Lourenço, M. A. M., Eckert, J. J. & Silva, L. C. A. Electric vehicle powertrain and fuzzy controller optimization using a planar dynamics simulation based on a real-world driving cycle. *Energy* **238**, 121979 (2022).

[40] Yuan, J. Q. & Zhang, L. Natural frequency and modal analysis of tractor vibration system. *Sci. Rep.* **15**, 1–13 (2025). 41006809 10.1038/s41598-025-18736-x PMC12474990

[41] Kim, W. S. et al. Traction performance evaluation of a 78-kW-class agricultural tractor using cone index map in a Korean paddy field. *J. Terramechanics*. **91**, 285–296 (2020).

[42] Baek, S. M. et al. Multi-objective macro-geometry optimization of a compound planetary geartrain for an electric tractor powertrain using NSGA-II. *Sci. Rep.* (2026). 10.1038/s41598-026-43864-3 PMC13247146 41998025

[43] Mujiburrahman, K., Saravanakumar, S., Satheesh kumar, K., Carciyo kaviya, J. J. & Krishnaraj, R. Design and analysis of E-glass gear box housing in tractor and optimization of its design parameters. *Mater. Today Proc.* (2021). 10.1016/j.matpr.2021.10.079

[44] Tang, J., Zhou, Z., Chen, H., Wang, S. & Gutiérrez, A. Research on the lightweight design of GFRP fabric pultrusion panels for railway vehicle. *Compos. Struct.* **286** (2022).

[45] Jacob, S., Thiruvarasan, V., Surendhar, S. & Senthamizh, R. Design, analysis and optimization of all terrain vehicle chassis ensuring structural rigidity. *Mater. Today Proc.* **46**, 3786–3790 (2020).

